# Further Investigation of the Dimensionality of the Questionnaire for Eudaimonic Well-Being

**DOI:** 10.3389/fpsyg.2022.795770

**Published:** 2022-05-06

**Authors:** Amanda Cromhout, Lusilda Schutte, Marié P. Wissing, Willem D. Schutte

**Affiliations:** ^1^Africa Unit for Transdisciplinary Health Research, Faculty of Health Sciences, North-West University, Potchefstroom, South Africa; ^2^Centre for Business Mathematics and Informatics, Faculty of Natural and Agricultural Sciences, North-West University, Potchefstroom, South Africa

**Keywords:** eudaimonic well-being, factorial validity, reliability, dimensionality, measurement invariance, bifactor ESEM

## Abstract

The dimensionality of the Questionnaire for Eudaimonic Well-Being (QEWB) has been a topic of debate and divergent findings in the literature up to date. This study investigated the factor structure and measurement invariance of the QEWB in four culturally diverse South African samples using confirmatory factor analysis (CFA), bifactor CFA, exploratory structural equation modelling (ESEM), and bifactor ESEM. Three student samples completed the English (*n* = 326), Afrikaans (*n* = 478), or Setswana (*n* = 260) version of the QEWB. An adult sample (*n* = 262) completed the English version. The one-factor structure revealed poor fit for the student samples. Although the four-factor models generally showed slightly better fit than the three-factor models, the latter was preferred for parsimony. The bifactor ESEM model displayed good fit for the student samples, with the general factor and some specific factors attaining sufficient reliability scores, pointing to the potential use of the scale in these samples. Configural invariance between the student samples was supported, but not metric nor scalar invariance. For the adult sample, none of the models displayed good fit and the use of the QEWB in this sample is not recommended. The results point towards the existence of a global eudaimonic well-being factor and, at the same time, the interrelatedness of facets of eudaimonic well-being. It suggests that eudaimonic well-being may be represented by the same items across the three student groups. The influence of developmental phase on the manifestation and measurement of eudaimonic well-being should be explored in future.

## Introduction

Eudaimonic well-being (EWB), together with hedonic well-being (HWB), are the main perspectives on well-being (Huta and Waterman, 2014) in the literature on psychosocial well-being, which is core in counselling theory and practice. Whereas HWB is mainly characterised by experiencing increased levels of positive emotions, reduced levels of negative emotions, and increased levels of life satisfaction (Diener, 1984; Waterman, 1993; Diener et al., 2017), EWB is conceptualised differently by different authors and generally includes reference to living or functioning well (see Martela and Sheldon, 2019). For example, Ryff (1989) discerned self-acceptance, personal growth, autonomy, positive relationships, environmental mastery, and purpose in life as elements of psychological well-being. The mental health continuum model (Keyes, 2002) specifies that the eudaimonic facet of positive mental health includes psychological well-being (as defined by Ryff, 1989) together with social well-being (comprising social coherence, social actualisation, social integration, social acceptance, and social contribution; Keyes, 1998). Martela and Sheldon (2019) indicated that at least 63 elements of EWB are used in about 45 operationalisations thereof. These include elements such as authenticity, emotional stability, mindfulness, optimism, resilience, and self-actualisation. All of these facets may be relevant in the enhancement of mental health, and the availability of valid and reliable measures based on sound theory is necessary to evaluate the outcomes of counselling interventions and growth. The conceptualisation and operationalisation of EWB by Waterman et al. (2010), which is described in the next paragraph, is relevant to this study.

### Conceptualisation of Eudaimonic Well-Being Informing the Questionnaire for Eudaimonic Well-Being

Waterman et al. (2010) postulated that EWB should be conceptualised based on the (then) current philosophical understandings of eudaimonic functioning, and discerned six interlinked categories which have strong associations with philosophy and psychology. The categories are: *self-discovery*, which is important for progression towards self-actualisation (and thus for experiencing EWB) and involves identifying who one is; *perceived development of one’s best potential*, which involves identifying and actively developing the unique potential that is representative of the best that one can become; *a sense of purpose and meaning in life*, which involves deciding towards which personally meaningful objectives one’s talents and skills will be directed; *investment of significant effort in pursuit of excellence*, which refers to individuals’ tendency to invest more effort in activities that they find personally meaningful than in other activities; *intense involvement in activities*, which refers to the intensity of the level of involvement in activities that individuals deem personally meaningful compared to their level of involvement in other activities; and *enjoyment of activities as personally expressive*, which refers to individuals’ involvement in activities that are expressive of who they are.

This conceptualisation of EWB includes both the objective and subjective elements of EWB (Waterman et al., 2010). The objective elements refer to the behaviours that are related to the pursuit of eudaimonic goals. The subjective elements refer to the experiences of individuals when they are committed to excellence in the actualisation of their personal potential. These subjective experiences of eudaimonia are called “feelings of personal expressiveness” and are typically associated with the pursuit of one’s life purpose and the development of one’s potential (Waterman et al., 2010, p. 42). Subjective feelings of personal expressiveness are different from subjective well-being (hedonia) in that the latter is a desired outcome in itself, while the former flows from the pursuit of life purpose and the development of potential (Waterman et al., 2010).

In order to test theoretical conceptualisations of EWB, to measure facets and levels of EWB, and to evaluate interventions aimed at enhancing EWB, it is important that psychometrically sound measures are used for this purpose. The Questionnaire for Eudaimonic Well-Being (QEWB; Waterman et al., 2010) is one measure of EWB and has been applied in several recent studies (e.g., Karaś and Chieciuch, 2018; Kimiecik et al., 2019; Sotgiu, 2019).

### The Questionnaire for Eudaimonic Well-Being

The Questionnaire for Eudaimonic Well-being (Waterman et al., 2010) measures EWB in terms of the conceptualisation of EWB by Waterman et al. (2010). Although six interlinked categories are discerned in this conceptualisation (Waterman et al., 2010), scale items were not assigned to the specific categories when the scale was constructed (Schutte et al., 2013; Klym-Guba and Karaś, 2018). For the purpose of scale construction, EWB was considered to be a unidimensional construct, where the six categories represent aspects of EWB (Klym-Guba and Karaś, 2018). Waterman et al. (2010) used parcelling and found support for a unifactorial structure in two ethnically diverse American student samples with Cronbach’s alpha values of 0.86 and 0.85, respectively. They also found support for convergent, discriminant, construct, and incremental validity. However, Schutte et al. (2013) questioned the use of parcelling and contended that the unidimensionality assumption within parcels was not tested and was likely not to have held. Applying confirmatory factor analysis (CFA) and exploratory factor analysis (EFA) to data from a multicultural South African student group, they found support for a three-factor structure [Sense of Purpose (Cronbach’s α = 0.77), Purposeful Personal Expressiveness (Cronbach’s α = 0.73), Effortful Engagement (Cronbach’s α = 0.61)] and a four-factor structure [Sense of Purpose (Cronbach’s α = 0.77), Engagement in Rewarding Activities (Cronbach’s α = 0.51), Living from Beliefs (Cronbach’s α = 0.71), Effortful Engagement (Cronbach’s α = 0.61)], thereby pointing towards the multidimensionality of the QEWB. Support was found for convergent and discriminant validity. Schutte et al. (2013) suggested that, although the four-factor solution explained slightly more variance than the three-factor solution, the three-factor solution was preferable in their sample for the sake of parsimony.

Subsequently, both the unidimensionality and the multidimensionality of the QEWB have been supported in recent studies. Applying CFA, Areepattamannil and Hashim (2017) found support for the unidimensionality of the QEWB in an Indian adolescent sample, reporting a Cronbach’s alpha value of 0.87. Sotgiu et al. (2019) applied Rasch-analysis to the Italian version of the QEWB in an Italian adult sample and also found support for a unidimensional structure. They reported a separation reliability *R* of 0.78 and a Cronbach’s alpha value of 0.81. Fadda et al. (2017) indicated that a unidimensional structure did not fit the data of the Italian version of the QEWB in Italian student samples. Applying bifactor ESEM to the three- and four-factor solutions found by Schutte et al. (2013), Fadda et al. (2017) found that the three-factor solution with one general EWB factor revealed superior fit. They reported model-based omega coefficients of composite reliability, namely general EWB factor (ω = 0.90), Sense of Purpose (ω = 0.97), Purposeful Personal Expressiveness (ω = 0.12), and Effortful Engagement (ω = 0.73), with scores on the general EWB factor correlating as expected with scores on measures of life satisfaction and self-esteem. In a subsequent study, Fadda et al. (2020) applied ESEM and bifactor ESEM to the Spanish version of the QEWB in a Spanish student sample, and found that the three-factor bifactor ESEM model outperformed the three-factor ESEM model. They reported sufficient levels of composite reliability with omega values of 0.97 for the general EWB factor, 0.84 for Sense of Purpose, 0.94 for Purposeful Personal Expressiveness, and 0.93 for Effortful Engagement. The general EWB factor correlated positively with a measure of self-esteem, while the specific factors showed no correlation with self-esteem. Applying CFA, EFA, and ESEM to the Polish translation of the QEWB, Klym-Guba and Karaś (2018) found that the three-factor ESEM model, with the three factors as distinguished by Schutte et al. (2013), adequately fitted the data. They reported Cronbach’s alpha values for the general EWB factor (α = 0.71 to 0.86), Sense of Purpose (α = 0.79 to 0.87), Purposeful Personal Expressiveness (α = 0.80 to 0.82), and Effortful Engagement (α = 0.63 to 0.71). Ishii et al. (2022) applied ESEM and bifactor ESEM to the Japanese translation of the QEWB in Japanese samples in different age groups (18–29; 30–49; and 50–69) and found that a four-factor ESEM model was most interpretable for the 18- to 29-year group, while a three-factor ESEM model was most interpretable for the 30- to 49-year group and the 50- to 69-year groups. For all groups the models included the Sense of Purpose, Purposeful Personal Expressiveness, and Effortful Engagement factors. Additionally, a “Deep and Meaningful Engagement” factor was discerned for the 18- to 29-age group.

Note that previous validation studies mostly used student samples (Waterman et al., 2010; Schutte et al., 2013; Fadda et al., 2017, 2020), except Areepattamannil and Hashim (2017) who used an adolescent sample and Ishii et al. (2022) who used Japanese adults in various age groups. Although Klym-Guba and Karaś (2018) and Sotgiu et al. (2019) described their samples as adult samples, the mean age of the adult samples used by Klym-Guba and Karaś (2018) was between 20 and 24 years of age across four samples, which is close to the mean age of the student groups used by Fadda et al. (2017, mean age 20 years), Fadda et al. (2020, mean age 20 years), and Schutte et al. (2013, mean age 21 years); and half of the adult sample used by Sotgiu et al. (2019) with a mean age of 28 years, consisted of students. Effectively, the study by Ishii et al. (2022) is the only study, as far as we could establish, that used mature adult samples to investigate the factor structure of the QEWB. The observation that studies exploring the psychometric properties of the QEWB among adults are limited is particularly important since EWB may be experienced differently across developmental phases. For example, Ryff and Keyes (1995) found in an adult sample, divided into young adults (25–29 years), midlife adults (30–64 years), and older adults (65 years and older), that there were differences among the age groups with regard to purpose in life, personal growth, environmental mastery, autonomy, self-acceptance, and personal relationships. Clarke et al. (2000) found that Canadian older adults (65 years or older) were likely to report a decline in their sense of environmental mastery, personal growth, purpose in life, and positive relationships with others with increasing age.

The measurement invariance of the QEWB has been explored in a few earlier studies. Areepattamannil and Hashim (2017) found support for gender invariance of the QEWB for an Indian adolescent sample. Fadda et al. (2020) also found that the Spanish version of the QEWB was gender invariant in a Spanish student sample. Sotgiu et al. (2019) found that the item measures obtained through Rasch analysis were gender invariant, but not age invariant, for the Italian version of the QEWB in an Italian adult sample. Klym-Guba and Karaś (2018) found support for the invariance of the Polish version of the QEWB across four young adult samples. As far as we could establish, no invariance studies investigated cross-cultural invariance of the scale. This is significant, since culture is fundamental to human behaviour, and should be key to theoretical and empirical investigations of psychological constructs (Matsumoto and Yoo, 2006), including eudaimonic well-being.

Besides the possibility that EWB may manifest differently from culture to culture or across sociodemographic different groups, which may influence the psychometric properties of measures of EWB, the statistical analytical procedures used to explore the dimensionality of a scale can also potentially influence the results. This aspect is addressed in the next paragraph.

### Measuring Multidimensional Constructs: Application of Exploratory Structural Equation Modelling and Bifactor Modelling

If statistical analyses that do not account for sources of multidimensionality are applied to model multidimensional constructs, it may result in biased parameter estimates (e.g., Morin et al., 2016a; Howard et al., 2018). For example, CFA is based on the independent cluster model (ICM) that assumes that the cross-loadings of items on non-target factors are exactly zero. However, when cross-loadings are constrained to zero, two sources of construct-relevant multidimensionality may not be accounted for, which may lead to biased parameter estimates (Morin et al., 2016b).

Firstly, scale items are rarely related to a single construct (the target factor) when a scale measures conceptually related constructs and will mostly also have construct-relevant associations with the non-target factors (Howard et al., 2018). When these cross-loadings are disregarded it may impact negatively on goodness-of-fit indices since sources of misspecification may be concealed. The discriminant validity of the factors may also be compromised when artificial multicollinearity is created by biased parameter estimates, and the factors are used in prediction (Howard et al., 2018). In order to account for these cross-loadings, exploratory structural equation modelling (ESEM, Asparouhov and Muthén, 2009) can be applied. With ESEM, EFA is incorporated into the structural equation modelling framework, which allows for models to be specified according to CFA specifications (thus accounting for target factor loadings), while also accounting for cross-loadings (Morin et al., 2016a; Howard et al., 2018).

Secondly, the scale items used to assess multiple dimensions in a psychometric measure could possibly reflect their specific subscales and more global constructs (Morin et al., 2016a). In such instances, hierarchical (or higher-order) CFA is typically applied (Morin et al., 2016a). Higher-order models hypothesise that multiple factors can combine into one or more higher-order factors. The model is specified by allowing each item to load on its specific subscale (i.e., first-order factor) and each first-order factor to load on a higher-order factor (Morin et al., 2016a). The first-order factor fully mediates the associations between the scale items and the higher-order factor (Morin et al., 2016b; Howard et al., 2018). The first-order factor therefore reflects the variance explained by each first-order factor and the variance explained by the higher-order factor (Morin et al., 2016b). In contrast, bifactor models hypothesise that a unitary global factor, that coexists with some specific factors, directly influences the scale items. The variance that is shared by all the scale items is represented by the global factor and the variance that is shared by a specific subset of scale items is represented by the specific factors (Morin et al., 2016b; Howard et al., 2018). The variance that is attributable to the global and specific factors, respectively, can therefore be separated, while simultaneously estimating the direct relations between scale items and the global and specific factors (Morin et al., 2016b; Howard et al., 2018).

Models that allow for the incorporation of cross-loadings and/or a general factor may display superior fit when constructs are conceptually related and/or hierarchically ordered. This is because the estimates of the global factor may be inflated when cross-loadings are not modelled in bifactor CFA models, and estimates of the cross-loadings may be inflated when the global factor is not modelled in EFA models (Morin et al., 2016a; Howard et al., 2018). Therefore, models like ESEM, bifactor CFA, and bifactor ESEM (Jennrich and Bentler, 2011) can be used.

As explicated in the previous section, the dimensionality of the QEWB has been a contentious issue in the literature up to date, with diverse findings being presented in different studies. In attempts to gain more insight into the dimensionality of the scale, ESEM has been applied to Polish (Klym-Guba and Karaś, 2018) and ESEM and bifactor ESEM to Italian (Fadda et al., 2017) and Spanish (Fadda et al., 2020) samples. All of these samples were European and consisted of students or young adults. More recently, ESEM and bifactor ESEM have also been applied to Japanese (Eastern) adult samples (Ishii et al., 2022). Extending the investigations to other cultural and age groups will provide insight into the dimensionality and manifestations of EWB.

### The Present Study

Newer analytical approaches, such as ESEM and bifactor ESEM, can provide insight into the dimensionality of a scale – a matter of particular importance for the QEWB for which divergent findings regarding its dimensionality have been presented in the literature. These methods have been applied to data from European (Polish, Italian, and Spanish) student or young adult samples (Fadda et al., 2017, 2020; Klym-Guba and Karaś, 2018), as well as to Eastern (Japanese) adult samples (Ishii et al., 2022). Since culture may largely influence the way in which psychological constructs such as eudaimonic well-being operate and manifest, it would be important to extend explorations to other, particularly non-Western, contexts. Notably, as far as we could establish no studies have investigated the cross-cultural measurement invariance of the scale. In addition, while age and developmental phase may impact how eudaimonic well-being is experienced and expressed, investigations on the psychometric properties of the QEWB have been done mostly on student or young adult samples. In view of these gaps, the aim of the present study was to provide a substantive illustration of various analytical models, namely CFA, bifactor CFA, ESEM, and bifactor ESEM models, to investigate the dimensionality of the QEWB in four culturally diverse South African samples (three student samples, one adult sample) who completed different language versions of the scale and to investigate measurement invariance across samples with adequate baseline fit.

## Materials and Methods

### Research Design and Participants

A quantitative, cross-sectional survey design was used. Three non-probability student samples (*N* = 1064) from the various campuses of a South African university completed the research battery in English (Sample 1, *n* = 326), Afrikaans (Sample 2, *n* = 478), or Setswana (Sample 3, *n* = 260). Participants could complete the research battery in their home language, or alternatively in the language they were most comfortable with. Participants who indicated “other” as their home language likely spoke one of the other 11 official languages of South Africa. Setswana is an indigenous African language, and participants who completed this version of the scale were most probably of indigenous African heritage. Afrikaans is a language close to Dutch, and taking this together with the demographic profile of the institution where data were gathered into consideration, the cultural heritage of participants who completed the Afrikaans scale version was probably strongly influenced by Western culture. Of the sample who completed the English version of the scale, 18.7% indicated that Setswana was their home language, while 54.9% picked “other.” This suggests that the sample was culturally diverse, but with the majority of participants having an African heritage. Sample 4 was a multicultural non-probability adult sample (*n* = 262) that was recruited with the snowball method across South Africa. The research battery was completed in English.

All samples had to be 18 years of age or older and have at least a Grade 12 level of education. Additionally, Samples 1, 2, and 3 had to be enrolled as students at the university where the data was collected. The socio-demographic information of participants from each sample is presented in Table 1.

**TABLE 1:** Socio-demographic profile of participants.

Variable	Sample 1	Sample 2	Sample 3	Sample 4
***n***	326	478	260	262
Gender				
Male	24.5%	35.8%	32.7%	33.2%
Female	75.5%	64.2%	67.3%	66.4%
Missing	0.9%	0%	0%	0.4%
*M*_*age*_ (*SD*_*age*_)	21.03 (4.08)	19.79 (3.14)	21.59 (4.59)	40.23 (12.19)
**Home language**				
English	18.4%	0.4%	21.9%	17.2%
Afrikaans	6.7%	99.2%	0.8%	32.4%
Setswana	18.7%	0%	66.5%	18.7%
Other	54.9%	0.4%	9.6%	14.5%
Missing	1.2%	0%	1.2%	17.2%
**Education level*^a^* (Sample 4)**				
Secondary	–	–	–	36.3%
Tertiary	–	–	–	32.4%
Post-graduate	–	–	–	29.8%
Missing	–	–	–	1.5%

*^a^Since Samples 1, 2, and 3 consisted of university students, education level was not assessed for this sample.*

*M, mean; SD, standard deviation.*

### Measures

#### Socio-Demographic Questionnaire

Data on socio-demographic variables such as age, gender, home language, and level of education (the latter for Sample 4) were collected.

#### The Questionnaire for Eudaimonic Well-Being

The QEWB (Waterman et al., 2010) consists of 21-items and measures EWB as conceptualised by Waterman et al. (2010). We used a seven-point Likert-type scale, ranging from 1 (*strongly disagree*) to 7 (*strongly agree*). Refer to the Introduction for detail on the scale development and previous findings on the psychometric properties of the scale.

### Ethical Considerations and Procedure

This study was approved by the Health Research Ethics Committee of the North-West University, South Africa (ethics approval number: NWU 00002-07-A2), and formed part of the FORT3 research project [The prevalence of levels of psychosocial health: Dynamics and relationships with biomarkers of (ill) health in South African social contexts; Wissing, 2008/2012]. Participants gave written informed consent, participated voluntarily in the study, and could withdraw from the study without adverse consequences. Data were handled confidentially, and participants received no incentives for participation.

The data of Samples 1, 2, and 3 were collected during 2012, and the data for Sample 4 were gathered during 2011–2014. For Samples 2 and 3, the QEWB was translated from English into Afrikaans and Setswana, respectively, using a research committee approach (Brislin, 1970; Van de Vijver and Humbleton, 1996; Van de Vijver and Leung, 1997). Scale items were checked for cultural appropriateness. The scale was back-translated into English by independent translators (Brislin, 1970). A research committee, that consisted of academics who spoke Afrikaans or Setswana natively and who were fluent in English, compared the back-translated and original English versions of the scale (Van de Vijver and Humbleton, 1996; Van de Vijver and Leung, 1997). A small pilot sample was asked to determine if the scale items of the translated versions were comprehensible and reflected the meaning of the items in a culturally appropriate manner, as well as to evaluate technical aspects such as the clarity of the format and layout of the research battery.

### Data Analysis

#### Stage 1: Descriptive Statistics of Individual Scale Items

IBM SPSS Statistics 25 was used to calculate the mean, standard deviation, and the univariate skewness and kurtosis of each item of the QEWB for all samples. The psych package (v2.1.9; Revelle, 2021) in R4.0.2 (R Core Team, 2021) was used to calculate Mardia’s multivariate skewness and kurtosis statistics.

#### Stage 2: Factorial Validity

All findings reported for factor analysis were based on analyses done using Mplus Version 8.3 (Putnick and Bornstein, 2017), unless otherwise specified. For all samples, the following models were tested: a one-factor CFA model, as well as the following three- and four-factor models: CFA, bifactor CFA, ESEM, and bifactor ESEM. The three- and four-factor models were based on the factors obtained by Schutte et al. (2013) when they performed exploratory factor analysis (EFA) on data from the scale. We used the robust maximum likelihood (MLR) estimator and applied full information likelihood estimation to handle missing data. For the CFA and bifactor CFA models the cross-loadings were constrained to zero, and for the ESEM and bifactor ESEM models cross-loadings were estimated to be close to, but not exactly, zero. We applied oblique target rotation to the ESEM models and orthogonal target rotation to the bifactor ESEM models (Asparouhov and Muthén, 2009). For both oblique and orthogonal rotations, factor variances were set to one, and for the orthogonal rotation, the factor covariances were set to zero (Asparouhov and Muthén, 2009). The following model fit statistics are reported: the χ^2^-statistic, comparative fit index (CFI), Tucker-Lewis index (TLI), root mean square error of approximation (RMSEA), and the standardised root mean square residual (SRMR). For the χ^2^-statistic, higher *p*-values indicate a closer fit between the hypothesised model and perfect fit (Bollen, 1989; Byrne, 2012). CFI and TLI values closer to 0.95 are representative of good model fit (Hu and Bentler, 1999; Byrne, 2012). RMSEA values smaller than 0.05 represent good model fit, while values up to 0.08 represent reasonable model fit (Byrne, 2012). SRMR values of 0.05 or less represent a well-fitting model (Byrne, 2012). The χ^2^-statistic is highly sensitive to sample size, therefore the CFI, TLI, RMSEA, and SRMR were used to interpret model fit. If the best-fitting model displayed inadequate fit, this model was used as the model from which areas of local misfit was explored (Byrne, 2012). Model misfit was identified by considering modification indices (MI) and the expected parameter change (EPC) values, where higher MI and EPC values point towards potential model misfit (Byrne, 2012; Whittaker, 2012). Although MI and EPC values were used to identify areas of misspecification, models were only modified if the changes also made sense on substantive grounds (Byrne, 2012; Whittaker, 2012).

Note that the unbiased SRMR fit index (derived by Maydeu-Olivares, 2017) was also calculated for the CFA and bifactor CFA models due to its superiority to other fit statistics (see Ximénez et al., 2022) using the lavResiduals function of the lavaan package (v0.6-10; Rosseel, 2012) in R4.0.2 (R Core Team, 2021). However, since fitting ESEM and bifactor ESEM models using lavaan is still in its infancy, the unbiased SRMR was not calculated for these models. In terms of interpretation, Shi et al. (2018) proposed that the unbiased SRMR divided by the average *R*^2^ of the items (denoted by $\bar{R^{2}}$) should be less than 0.05 for models with an acceptable fit.

#### Stage 3: Internal Consistency Reliability

Microsoft Excel was used to calculate model-based omega coefficients of composite reliability, using the formula applied by Sánchez-Oliva et al. (2017). The formula is


$$\omega =\sum {\left(\left|\lambda_{i}\right|\right)}^{2}/\left(\sum {\left[\left|\lambda_{i}\right|\right]}^{2}+\sum \delta_{i\,i}\right),$$

where the factor loadings are represented by λ_*i*_, and the error variances by δ_*ii*_ (McDonald, 1970). Calculations were based on parameter estimates obtained from Mplus output. According to Putnick and Bornstein (2017), the guideline that reliability scores larger than 0.70 or 0.80 indicate acceptable reliability is not suitable for bifactor models (see Putnick and Bornstein, 2017, for an explanation). Instead they suggest that omega values larger than 0.50 are indicative of sufficient reliability for bifactor models.

#### Stage 4: Measurement Invariance

Mplus Version 8.3 (Putnick and Bornstein, 2017) was used to determine invariance across the different language versions of the QEWB in student Samples 1, 2, and 3 (Sample 4 was not included in invariance analyses, since no baseline model with adequate fit could be obtained). We tested for configural, metric, and scalar invariance (Morin et al., 2016a; Putnick and Bornstein, 2017). No equality constraints are applied when testing for configural invariance (Byrne, 2012). If the factor loadings display the same pattern across the groups, configural invariance is supported (Putnick and Bornstein, 2017). For metric and scalar invariance equality constraints are applied. Factor loadings are constrained to be equivalent across the groups for metric invariance, and factor loadings and intercepts in the case of scalar invariance. If metric or scalar invariance is not supported, the non-equivalent factor loadings and intercepts can be released in order to establish support for partial metric or partial scalar invariance (Putnick and Bornstein, 2017). Non-equivalent factor loadings and intercepts can be identified by considering high MI and EPC values (Byrne, 2012). Differences smaller than 0.01 and 0.015 between the CFI and RMSEA values of the nested models, respectively, indicate measurement invariance (Cheung and Rensvold, 2002; Chen, 2007). The likelihood ratio test, which is based on the difference between the χ^2^-statistic of the nested models, is highly sensitive to sample size (Cheung and Rensvold, 2002; Chen, 2007). We reported the results of this test but placed more emphasis on other indicators for decision-making.

## Results

### Stage 1: Descriptive Statistics of Individual Scale Items

For Sample 1 mean values ranged between 3.99 (*SD* = 2.03; item 3) and 6.28 (*SD* = 1.21; item 15), skewness values ranged between −2.35 (item 15) and 0.4 (item 3), and kurtosis values ranged between −1.37 (item 16) and 6.42 (item 15) for the QEWB-English. For Sample 2 mean values ranged between 4.07 (*SD* = 1.70; item 9) and 6.01 (*SD* = 1.30; item 19), skewness values ranged between −1.73 (item 19) and −0.19 (items 9 and 16), and kurtosis values ranged between −0.98 (item 3) and 2.97 (item 19) for the QEWB-Afrikaans. For Sample 3 mean values ranged between 3.25 (*SD* = 2.05; item 3) and 5.78 (*SD* = 1.39; item 18), skewness values ranged between −1.31 (item 5) and 0.15 (item 20), and kurtosis values ranged between −1.36 (item 20) and 1.29 (item 6) for the QEWB-Setswana. For Sample 4 mean values ranged between 3.83 (*SD* = 1.98, item 3) and 5.89 (*SD* = 1.25; item 15), skewness values between −1.38 (item 15) and 0.20 (item 3), and kurtosis values between −1.15 (item 20) and 2.18 (item 15) for the QEWB-English.

There was deviation from normality in Sample 1 as indicated by a few skewness and kurtosis values that were in absolute value larger than 2 and in Samples 2 and 4 as indicated by some kurtosis values that were in absolute value larger than 2 (Bandalos and Finney, 2010). For Sample 3 all skewness and kurtosis values were in absolute value smaller than 2 (Bandalos and Finney, 2010). For all samples, the *p*-values of the test statistics of Mardia’s multivariate skewness and kurtosis were small, pointing to deviations from multivariate normality. The descriptive statistics of the individual scale items for all samples are presented in Supplementary Table 1, the multivariate skewness and kurtosis values in Supplementary Table 2, and the inter-item correlations are presented in Supplementary Tables 3–6.

### Stage 2: Factorial Validity

**FIGURE 1:**
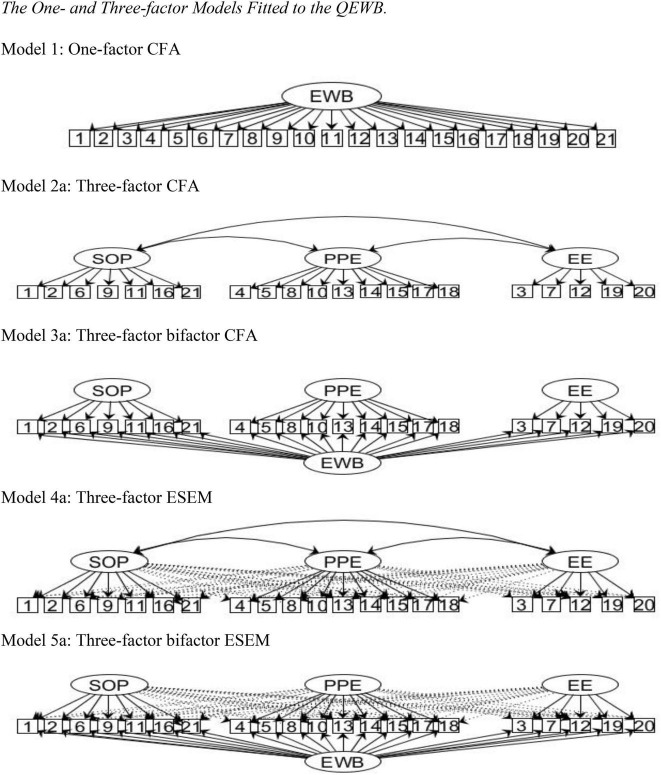
The one- and three-factor models fitted to the QEWB. Model 1: One-factor CFA. Model 2a: Three-factor CFA. Model 3a: Three-factor bifactor CFA. Model 4a: Three-factor ESEM. Model 5a: Three-factor bifactor ESEM. EWB, Eudaimonic Well-being; SOP, Sense of Purpose factor; PPE, Purposeful Personal Expressiveness factor; EE, Effortful Engagement factor.

**FIGURE 2:**
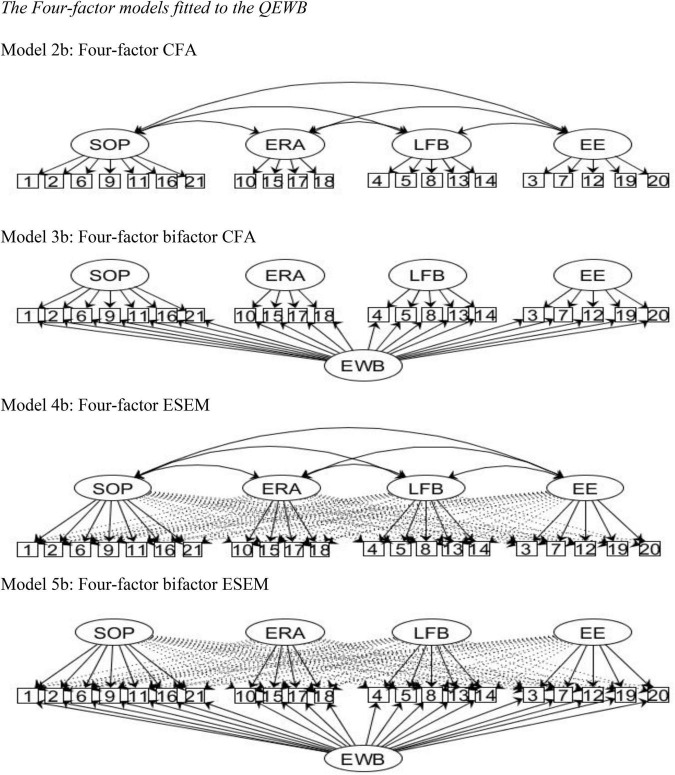
The four-factor models fitted to the QEWB. Model 2b: Four-factor CFA. Model 3b: Four-factor bifactor CFA. Model 4b: Four-factor ESEM. Model 5b: Four-factor bifactor ESEM. EWB, Eudaimonic Well-being; SOP, Sense of Purpose factor; ERA, Engagement in Rewarding Activities; LFB, Living from Beliefs; EE, Effortful Engagement factor.

The various models tested for the four samples are portrayed in Figures 1, 2: a one-factor CFA (Model 1); and the following three- and four-structure models: CFA (Models 2a and 2b), bifactor CFA (Models 3a and 3b), ESEM (Models 4a and 4b), and bifactor ESEM (Models 5a and 5b). The fit indices are presented in Table 2. Model 1 revealed poor fit for all samples. Models 4 showed improved fit indices compared to Models 2, while Models 3 and 5 fitted better than Models 2 and 4. Although the four-factor models yielded slightly improved fit indices compared to the three-factor models, we preferred the three-factor structure for the sake of parsimony. The focus of this section will henceforth be on reporting the detailed results for analyses done using the three-factor structure.

**TABLE 2:** Fit indices for the one-, three-, and four-factor models.

Latent model	χ^2^	*df*	*p*	CFI	TLI	RMSEA	90% CI of RMSEA	SRMR	SRMR_*u*_	90% CI of SRMR_*u*_	${\text{SRMR}}_{\text{u}}/\bar{R^{2}}$
**Sample 1: QEWB-English (students)**											
*1-factor*											
Model 1	560.472	189	<0.001	0.631	0.590	0.078	(0.070; 0.085)	0.087	0.071	(0.055; 0.087)	0.349
*3-factor*											
Model 2a	383.762	186	<0.001	0.804	0.778	0.057	(0.049; 0.065)	0.074	0.052	(0.037; 0.067)	0.178
Model 3a*^a^*	323.438	168	<0.001	0.846	0.807	0.053	(0.045; 0.062)	0.057	0.029	(0.017; 0.042)	0.091
Model 4a	246.033	150	<0.001	0.905	0.866	0.044	(0.034; 0.054)	0.043	N/A	N/A	N/A
Model 5a	180.810	132	0.0031	0.952	0.923	0.034	(0.020; 0.045)	0.036	N/A	N/A	N/A
*4-factor*											
Model 2b	370.108	183	<0.001	0.814	0.787	0.056	(0.048; 0.064)	0.073	0.049	(0.034; 0.064)	0.156
Model 3b*^a^*	333.162	168	<0.001	0.836	0.795	0.055	(0.046; 0.064)	0.062	0.026	(0.014; 0.039)	0.078
Model 4b	180.810	132	0.0031	0.952	0.923	0.034	(0.020; 0.045)	0.036	N/A	N/A	N/A
Model 5b	147.231	115	0.0229	0.968	0.942	0.029	(0.012; 0.043)	0.030	N/A	N/A	N/A
**Sample 2: QEWB-Afrikaans (students)**											
*1-factor*											
Model 1	599.253	189	<0.001	0.748	0.720	0.067	(0.061; 0.073)	0.068	0.060	(0.05; 0.07)	0.295
*3-factor*											
Model 2a	392.096	186	<0.001	0.873	0.857	0.048	(0.041; 0.055)	0.059	0.046	(0.037; 0.056)	0.164
Model 3a*^a^*	274.518	168	<0.001	0.935	0.918	0.036	(0.028; 0.044)	0.043	0.023	(0.016; 0.031)	0.074
Model 4a	219.893	150	<0.001	0.957	0.940	0.031	(0.022; 0.040)	0.031	N/A	N/A	N/A
Model 5a	171.566	132	0.0117	0.976	0.961	0.025	(0.012; 0.035)	0.027	N/A	N/A	N/A
*4-factor*											
Model 2b*^a,b^*	378.854	183	<0.001	0.880	0.862	0.047	(0.041; 0.054)	0.057	0.044	(0.035; 0.054)	0.160
Model 3b*^a^*	No convergence in Mplus								0.024	(0.017; 0.031)	0.079
Model 4b	171.566	132	0.0117	0.976	0.961	0.025	(0.012; 0.035)	0.027	N/A	N/A	N/A
Model 5b	153.891	115	0.0090	0.976	0.956	0.027	(0.014; 0.037)	0.024	N/A	N/A	N/A
**Sample 3: QEWB-Setswana (students)**											
*1-factor*											
Model 1	505.915	189	<0.001	0.635	0.594	0.081	(0.072; 0.089)	0.090	0.070	(0.051; 0.089)	0.364
*3-factor*											
Model 2a	420.470	186	<0.001	0.730	0.695	0.070	(0.061; 0.079)	0.086	0.068	(0.052; 0.084)	0.275
Model 3a*^a^*	No convergence in Mplus								0.017	(0.002; 0.032)	0.059
Model 4a*^c^*	146.249	133	0.204	0.983	0.975	0.020	(0.000; 0.037)	0.038	N/A	N/A	N/A
Model 5a*^c^*	134.157	116	0.119	0.976	0.961	0.025	(0.000; 0.041)	0.031	N/A	N/A	N/A
*4-factor*											
Model 2b	412.410	183	<0.001	0.736	0.697	0.070	(0.061; 0.079)	0.086	0.063	(0.047; 0.079)	0.242
Model 3b*^a^*	No convergence in Mplus								0.022	(0.008; 0.036)	0.071
Model 4b*^c^*	134.157	116	0.119	0.976	0.961	0.025	(0.000; 0.041)	0.031	N/A	N/A	N/A
Model 5b*^d^*	60.390	73	0.854	1.000	1.043	0.000	(0.000; 0.021)	0.023	N/A	N/A	N/A
**Sample 4: QEWB-English (adults)**											
*1-factor*											
Model 1	891.433	189	<0.001	0.400	0.333	0.119	(0.111; 0.127)	0.124	0.100	(0.081; 0.118)	0.510
*3-factor*											
Model 2a	693.079	186	<0.001	0.567	0.511	0.102	(0.094; 0.110)	0.116	0.094	(0.072; 0.117)	0.327
Model 3a	No convergence in Mplus or lavaan										
Model 4a	324.457	150	<0.001	0.851	0.791	0.067	(0.057; 0.077)	0.050	N/A	N/A	N/A
Model 5a	281.429	132	<0.001	0.872	0.797	0.066	(0.055; 0.076)	0.045	N/A	N/A	N/A
*4-factor*											
Model 2b	679.200	183	<0.001	0.576	0.513	0.102	(0.094; 0.110)	0.114	0.083	(0.063; 0.103)	0.279
Model 3b	No convergence in Mplus or lavaan										
Model 4b	281.429	132	<0.001	0.872	0.797	0.066	(0.055; 0.076)	0.045	N/A	N/A	N/A
Model 5b	276.883	115	<0.001	0.862	0.747	0.073	(0.062; 0.084)	0.041	N/A	N/A	N/A

*QEWB, Questionnaire for Eudaimonic Well-Being; **1-factor:** Model 1 = confirmatory factor analysis (CFA); **3-factor and 4-factor:** Model 2 = CFA, Model 3 = bifactor CFA; Model 4 = exploratory structural equation modelling (ESEM); Model 5 = bifactor ESEM; χ^2^, Chi-square; df, degrees of freedom; p, probability value; CFI, comparative fit index; TLI, Tucker–Lewis index; RMSEA, root mean square error of approximation; 90% CI of RMSEA, 90% confidence interval of the RMSEA; SRMR, standardised root mean square residual; SRMR_u_, unbiased SRMR calculated using lavaan; 90% CI of SRMR_u_ = 90% confidence interval of the SRMR_u_; ${\text{SRMR}}_{\text{u}}/\bar{R^{2}}$ SRMR_u_ divided by the average R^2^ of the items. N/A indicates that the SRMR_u_ was not calculated for the ESEM or bifactor ESEM models. All fit statistics were calculated using Mplus Version 8.3 except for the SRMR_u_ that was calculated using the lavResiduals function of the lavaan package in R4.0.2.*

*^a^Lavaan output in R warns that variance-covariance matrix does not appear to be positive definite.*

*^b^Mplus output warns that Psi matrix is not positive definite.*

*^c^Item 9 removed (negative residual variance).*

*^d^Items 9, 1, 18 removed (negative residual variance).*

For Samples 1 and 2, Model 5a showed best fit. For Sample 3, Models 4a and 5a, with item 9 (‘‘I can say that I have found my purpose in life’’) removed, fitted best. Item 9 in the QEWB-Setswana had a negative residual variance^1^ which suggested removal of the item. Item 9 was removed on statistical grounds. Since Model 5a performed well in Samples 1 and 2 and Model 5a, with item 9 removed, performed well across Samples 1, 2, and 3, we selected these models for invariance testing. For Sample 4, all models tested revealed poor fit. Several attempts to find a model with better fit as suggested by high MI’s and EPC’s, or removing items with negative residual variances, while bearing in mind substantive considerations, did not produce any model with good fit that made substantive sense. We therefore concluded that we could not find support for the validity of the QEWB for Sample 4. The remainder of this section will present further results for Samples 1, 2, and 3.

Next, we examined the factor loadings of the items. The standardised factor loadings for the final preferred models of Samples 1, 2, and 3 (Model 5a for the QEWB-English [Sample 1] and QEWB-Afrikaans [Sample 2], and for the QEWB-Setswana [Sample 3] Model 5a with item 9 removed) are presented in Table 3. For Sample 1, all items had statistically significant loadings on the general factor. For the specific factors target factor loadings were generally larger than cross-loadings and were all statistically significant for the SOP and EE factors. For the PPE factor only item 4 had a statistically significant target factor loading, while items 8 and 13 had larger statistically significant cross-loadings on the SOP factor. The SOP and EE factors had target factor loadings that were generally larger than the loadings on the general factor. Target factor loadings on the PPE factor were generally smaller than the loadings on the general factor.

**TABLE 3:** Standardised factor loadings and omega coefficients for the final preferred 3-factor models of the QEWB (Samples 1, 2, and 3).

	Sample 1: QEWB-English				Sample 2: QEWB-Afrikaans				Sample 3: QEWB-Setswana			
	bifactor ESEM				bifactor ESEM				bifactor ESEM (item 9 out)			
			
Item	G	SOP	PPE	EE	G	SOP	PPE	EE	G	SOP	PPE	EE
***SOP factor***												
1	0.39*	0.26*	0.01	0.08	0.55*	**0.02**	0.14*	−0.08	0.35*	0.55*	−0.01	−0.26*
2	0.40*	0.59*	0.12	−0.04	0.66*	**0.20**	−0.08	−0.13*	0.48*	**0.28**	0.13	−0.12
6	0.53*	0.43*	−0.15	0.09	0.62*	**−0.11**	0.14	0.09	0.58*	**0.04**	0.06	−0.07
9	0.40*	0.64*	0.05	−0.10	0.68*	**0.38**	−0.03	−0.12	**–**	**–**	–	–
11 (R)	0.25*	0.46*	0.09	0.26*	0.35*	0.71*	0.05	0.29*	0.34*	**0.23**	−0.07	0.59*
16 (R)	0.15*	0.35*	−0.04	0.34*	0.43*	**0.20**	−0.19*	0.13*	**0.08**	0.32*	−0.01	0.41*
21	0.26*	0.61*	0.04	−0.15	0.69*	**0.39**	−0.06	−0.11	0.58*	**0.06**	−0.02	0.02
***PPE factor***												
4	0.60*	0.13	0.58*	−0.03	0.41*	0.00	0.18*	0.08	0.49*	0.13	**0.06**	−0.16*
5	0.47*	0.05	**0.16**	0.06	0.33*	−0.04	**0.20**	0.08	0.37*	−0.09	**−0.17**	0.08
8	0.58*	−0.11*	**0.00**	−0.05	0.36*	−0.10	**0.07**	0.05	0.51*	−0.16	**−0.19**	0.08
10	0.19*	−0.12	**−0.18**	−0.18	0.16*	0.02	0.25*	−0.12	0.32*	−0.13	**−0.21**	−0.02
13	0.63*	−0.19*	**0.15**	−0.09	0.35*	−0.04	0.39*	0.01	0.63*	0.04	**−0.04**	0.06
14	0.48*	0.08	**0.00**	0.00	0.45*	−0.01	0.32*	−0.06	0.52*	0.21*	**−0.15**	−0.26*
15	0.60*	0.11	**−0.33**	0.09	0.37*	−0.11	0.41*	0.03	0.57*	−0.10	**0.19**	0.05
17	0.47*	0.10	**−0.10**	−0.05	0.45*	0.02	0.42*	0.10	0.42*	0.13	**0.13**	0.02
18	0.61*	−0.20*	**−0.31**	−0.03	0.26*	0.00	0.62*	−0.06	0.66*	−0.08	**0.40**	0.03
***EE factor***												
3 (R)	**0.10**	−0.02	0.13	0.37*	0.18*	0.01	0.02	0.41*	**−0.23**	0.10	0.23	0.23*
7 (R)	0.24*	0.13	−0.11	0.41*	0.17*	0.05	0.01	0.36*	**0.21**	−0.05	0.22*	0.37*
12 (R)	0.24*	−0.03	−0.01	0.34*	0.31*	0.21*	0.17*	0.33*	0.30*	0.09	−0.10	0.57*
19 (R)	0.23*	−0.05	−0.03	0.59*	0.28*	−0.01	−0.07	0.41*	0.33*	0.10	−0.06	0.64*
20 (R)	0.28*	0.22*	0.03	0.50*	0.39*	0.06	−0.09	0.36*	**0.11**	−0.06	−0.03	0.45*
*Omega coefficients*	0.83	0.73	0.37	0.58	0.84	0.54	0.55	0.48	0.83	0.38	0.29	0.60

*QEWB, Questionnaire for Eudaimonic Well-Being; ESEM, exploratory structural equation modelling; G, general factor; SOP, Sense of Purpose factor; PPE, Purposeful Personal Expressiveness factor; EE, Effortful Engagement factor; (R), item is reverse scored. Factor loadings on the general factor and target factor loadings on the intended specific factors are indicated in bold. Scale items are available from Waterman et al. (2010).*

*p < 0.05.

For Sample 2, all items had significant loadings on the general factor. Only item 11 (belonging to the SOP factor) loaded significantly on the SOP factor. Except for items 5 and 8 of the PPE factor, all items loaded significantly on the specific target factor for the PPE and EE factors. All target factor loadings were larger than cross-loadings, except for SOP item 1 that had a larger statistically significant cross-loading on the PPE factor. Although specific factor loadings were mostly larger than 0.3, item loadings on the general factor were mostly larger than specific target factor loadings.

For Sample 3, all items, except items 3, 7, 16, and 20, had statistically significant loadings on the general factor. SOP items 1 and 16 had statistically significant target factor loadings on the SOP factor, while SOP items 11 and 16 had statistically significant cross-loadings on the EE factor. There were no statistically significant target factor loadings on the PPE factor, but items 4 and 14 had statistically significant cross-loadings on the EE factor. All EE items had statistically significant target factor loadings on the EE factor, with no statistically significant cross-loadings on non-target factors. For the SOP and PPE factors, loadings on the general factor were mostly larger than target factor loadings. For the EE factor target factor loadings were larger than loadings on the general factor. Although only a few target factor loadings, mostly that of the EE subscale, were larger than 0.3, target factor loadings were mostly larger than cross-loadings.

### Stage 3: Internal Consistency Reliability of the Final Preferred Models for Samples 1, 2, and 3

Omega coefficients for Samples 1, 2, and 3 are presented in Table 3. Support for reliability of scores of the general factor was established for all groups with ω-values higher than 0.50 (Putnick and Bornstein, 2017). Except for the PPE factor of the QEWB-English (Sample 1), the EE factor of the QEWB-Afrikaans (Sample 2), and the SOP and PPE factors of the QEWB-Setswana (Sample 3), support was established for reliability of the specific factor scores for the three student samples.

### Stage 4: Measurement Invariance

Model 5a was chosen as the final preferred model for Samples 1, 2, and 3, but item 9 had to be removed for Sample 3 who completed the QEWB-Setswana. To find a baseline model for testing measurement invariance, we first investigated the fit of Model 5a with item 9 removed to data from Samples 1 and 2. Good fit was obtained for Sample 1 (CFI = 0.964; RMSEA = 0.029) and Sample 2 (CFI = 0.969; RMSEA = 0.028). We therefore conducted two sets of measurement invariance tests: First, we tested measurement invariance between Samples 1 and 2 using Model 5a as baseline model. Then we tested measurement invariance between Samples 1, 2, and 3 using Model 5a with item 9 removed as baseline model. The results are presented in Table 4.

**TABLE 4:** Measurement invariance of the QEWB for Samples 1, 2, and 3.

Model	χ^2^	*df*	*p*	CFI	RMSEA	Model comparison	χ^2^	*df*	*p*	Δ CFI	Δ RMSEA
**Samples 1 and 2 (Model 5a)**											
Invariance Model 1	353.179	264	<0.001	0.966	0.029	–	–	–	–	–	–
Invariance Model 2A	485.643	332	<0.001	0.941	0.034	2A vs. 1	131.291	68	<0.001	−0.025	0.005
Invariance Model 2B	470.891	328	<0.001	0.945	0.033	2B vs. 1	114.807	64	0.000	−0.021	0.016
Invariance Model 2C	440.588	322	<0.001	0.954	0.030	2C vs. 1	86.199	58	0.010	−0.012	0.001
Invariance Model 2D	417.300	314	<0.001	0.960	0.029	2D vs. 1	64.553	50	0.081	−0.006	0.000
Invariance Model 3	No convergence										
**Samples 1, 2, and 3 (Model 5a, item 9 removed)**											
Invariance Model 1	472.511	348	<0.001	0.959	0.032	–	–	–	–	–	–
Invariance Model 2A	685.999	476	<0.001	0.930	0.035	2A vs. 1	206.723	128	<0.001	−0.029	0.003
Invariance Model 2B	The first-order derivative product matrix, as well as the latent variable covariance matrix for Sample 3 was not positive definite										

***Samples 1 and 2 (Model 5a):** Invariance Model 1 = configural invariance model; Invariance Model 2A = metric invariance model; Invariance Model 2B = partial metric invariance model with the factor loading of item 11 on the Effortful Engagement factor freely estimated in both groups; Invariance Model 2C = partial metric invariance model with the factor loadings of items 11 and 6 on the Effortful Engagement factor freely estimated in both groups; Invariance Model 2D = partial metric invariance model with the factor loadings of items 11 and 6 on the Effortful Engagement factor and item 8 on the Sense of Purpose factor freely estimated in both groups; Invariance Model 3 = partial scalar invariance model; **Samples 1, 2, and 3 (Model 5a, item 9 removed):** Invariance Model 1 = configural invariance model; Invariance Model 2A = metric invariance model; Model 2B = partial metric invariance model with the factor loading of item 3 on the Effortful Engagement factor freely estimated in all groups; χ^2^, Chi square; df, degrees of freedom; p, probability value; CFI, comparative fit index; TLI, Tucker–Lewis index; RMSEA, root mean square error of approximation; 90% CI, 90% confidence interval of the RMSEA; SRMR, standardised root mean square residual.*

For Samples 1 and 2 (using Model 5a as baseline model), the configural invariance model fitted the data well (CFI = 0.966; RMSEA = 0.029). When testing for metric invariance, several factor loadings had to be freely estimated in the two groups to reach a point where support for partial metric invariance was indicated by adequately small ΔCFI and ΔRMSEA values. Selection of parameters to free was based on relatively small MI-values (less than 10) and these changes were not substantively justifiable. The subsequent partial scalar invariance model did not converge. We therefore concluded that only support for configural invariance was established.

For Samples 1, 2, and 3 (using Model 5a with item 9 removed as baseline model), the configural invariance model yielded good fit (CFI = 0.959; RMSEA = 0.032). However, full metric invariance was not supported and when testing for partial metric invariance, the first-order derivative product matrix, as well as the latent variable covariance matrix for Sample 3 was not positive definite. We therefore concluded that only support for configural invariance was established.

## Discussion

This study explored the dimensionality of the QEWB in four culturally diverse South African samples (three student samples, one adult sample) who completed different language versions of the scale, demonstrating the performance of the scale when applying different analytic techniques. Measurement invariance was also examined where sufficient baseline fit was obtained. The bifactor ESEM model best fitted the data for all student samples, although item 9 had to be removed from the QEWB-Setswana. Although scale items should ideally be removed on both statistical and substantive grounds (Byrne, 2012), item 9 was removed on statistical grounds only as there were no clear substantive reasons for its removal. Future research may investigate whether this result replicates in other samples. For the student samples, support was established for the reliability of scores on the general EWB factor and some specific factors. None of the tested models fitted the adult sample. Configural invariance was supported between the student samples, but not metric or scalar invariance. Findings will be discussed in the paragraphs below.

### Dimensionality of the Questionnaire for Eudaimonic Well-Being

The results for student Samples 1, 2, and 3 are in line with research that supported the multidimensionality of the QEWB (e.g., Schutte et al., 2013; Fadda et al., 2017, 2020; Klym-Guba and Karaś, 2018; Salavera and Usán, 2019; Ishii et al., 2022). The one-factor CFA model showed poor model fit for all the student samples. Although the four-factor structure yielded models with slightly better fit compared to the three-factor models, we selected the more parsimonious three-factor structure for our final preferred models. The three-factor structure was also supported by Klym-Guba and Karaś (2018) who, with the application of ESEM, obtained similar item-factor fit as Schutte et al. (2013), except for items 1 and 6 that loaded on the PPE factor, item 4 that loaded on the SOP factor, and item 10 that was removed.

Our results point towards the multidimensionality of EWB and support the existence of a general EWB factor that coexists with some specific EWB factors. The results further point towards the limitations inherent in CFA and indicate that multidimensional constructs may be represented better by statistical models that account for sources of multidimensionality. Firstly, model fit improved when cross-loadings were modelled (e.g., ESEM models). The cross-loadings were generally small and can therefore be regarded as the influence of the non-target factor on the construct-relevant part of the item (Morin et al., 2016a). Small cross-loadings compared to loadings on target factors point towards the factorial validity of all the language versions of the QEWB for the student samples. Secondly, the improvement in model fit when a general factor was modelled (e.g., bifactor CFA and bifactor ESEM), indicates that a general EWB factor (that directly influences all items of the QEWB) coexists with the specific EWB factors. Together these results indicate that the inclusion of cross-loadings and/or a general factor resulted in improved model fit, thereby pointing towards the existence of a global eudaimonic well-being factor and the interrelatedness of the specific eudaimonic well-being factors.

### Measurement Invariance

We established support for configural invariance between Samples 1 and 2 when the three-factor bifactor ESEM model was applied; and between Samples 1, 2, and 3 when the three-factor bifactor ESEM model, with item 9 removed, was applied. This implies that the same factor structure of the QEWB held across the relevant samples (cf. Lee, 2018) and that latent theoretical constructs are associated with the same items, connoting that the same items can be used to measure the constructs across the groups (Boer et al., 2018). However, neither full nor partial metric or scalar invariance was established across the samples. The samples can therefore not be compared on factor variances and covariances, nor on factor mean scores. These findings are noteworthy because it means that, although factor loadings and factor mean scores cannot be compared, there are similarities in how eudaimonic well-being is experienced and expressed across the more African and more Western cultural groups.

### Measuring Eudaimonic Well-Being Across Age Groups

For Sample 4, the adult sample, none of the models tested displayed good fit. This could not be remedied by correlating residual variances of item pairs suggested by high MI and EPC values, nor by setting residual variances to be larger than zero to avoid negative residual variance values. This finding is in contrast with the good psychometric properties of the QEWB found in previous studies (Waterman et al., 2010; Schutte et al., 2013; Areepattamannil and Hashim, 2017; Fadda et al., 2017, 2020; Klym-Guba and Karaś, 2018), as well as in the student samples of the current study. In another study amongst adults, Ishii et al. (2022), that used Japanese adult samples, tested several different models with different factor structures before they selected the four-factor ESEM model for the 18- to 29-years age group and the three-factor ESEM model for the 30- to 49-year age group and the 50- to 69-year age group (see Ishii et al., 2022 for a description). The fit indices were inconsistent across the age groups, but suggested that three to five factors are most appropriate for the QEWB. The other models, including the bifactor models, did not yield interpretable results. The only other study of the QEWB’s psychometric properties amongst adults was by Sotgiu et al. (2019) who used a sample in a similar age range (18–60 years) to our adult sample (18–74 years). They applied Rasch analysis and found that a unidimensional factor structure displayed good model fit for the QEWB. However, with a mean age of 28 years for the sample in the study by Sotgiu et al. (2019) and a mean age of 40 years for our adult sample, as well as the fact that Sotgiu et al. (2019) applied Rasch-analysis, which is different to the statistical techniques applied in this study, the comparison of the results between our study and the study done by Sotgiu et al. (2019) with regard to model fit should be made with caution. Overall, it does seem as if the developmental phase of the participants may play a role in the psychometric performance of the QEWB.

The adult sample in this study consisted mainly of participants in young (18–40 years) and middle (40–65 years) adulthood as discerned by Erikson (1997). According to Erikson (1997) young adulthood is the developmental phase during which individuals become less self-directed as they become more concerned with the developmental task of forming intimate and long-term relationships with others. During middle adulthood the main developmental task is to develop generativity, which involves the concern to contribute to others and society by actions such as parenting, volunteering, mentoring, and engaging in productive and meaningful work (Erikson, 1997). Adults in midlife (40–65 years of age) search for meaning in life and may adapt their sense of identity reflecting on their lives so far (Kuther and Burnell, 2019). To the contrary, the student samples in this study (mean age between 19 and 21 years) are emerging adults (18–25 years, Arnett, 2000). During this phase the development of a sense of self is regarded as the main developmental task (Erikson, 1997). Although this stage was initially associated with adolescence (12–18 years, Erikson, 1997), it was later recognised that this stage may last into emerging adulthood (Arnett, 2000). Emerging adulthood is characterised by, *inter alia*, identity explorations (e.g., developing one’s identity through the exploration of various life possibilities), instability (e.g., experiencing life changes), self-focus (being focused on oneself while acquiring skills needed for adulthood), feeling in-between (e.g., subjectively experience that one is in a transitional phase of life), and possibilities/optimism (e.g., believing that the future holds possibilities; Arnett, 2004; Arnett and Mitra, 2018). These features were proposed to be more prominent in, but not exclusive to, emerging adulthood (Arnett, 2004; Arnett and Mitra, 2018), and may differ across cultures (Arnett, 2011).

The main developmental tasks associated with each developmental phase may have influenced how the student and adult samples, respectively, responded to the items. For example, items measuring “self-discovery” may have been more relevant in the student groups, while items measuring “sense of purpose and meaning in life” may have been more relevant in the adult group. In this regard, Sotgiu et al. (2019), who applied Rasch-analysis, indicated that certain items of the Italian version of the QEWB were more typical of some age groups than of other. They found that items 3 (“I think it would be ideal if things came easily to me in my life”), 12 (“I can’t understand why some people want to work so hard on the things that they do”), and 19 (“If something is really difficult, it probably isn’t worth doing”) were more typical of emerging adults (18–25 years), item 11 (“As yet, I’ve not figured out what to do with my life”) was more typical of young adults (26–35 years) and middle-aged adults (36–60 years), and items 2 (“I believe I have discovered who I really am”), 19 (“If something is really difficult, it probably isn’t worth doing”), and 21 (“I believe I know what I was meant to do in my life”) were more typical of middle-aged adults (36–60 years). They argued that EWB seemed to have been cultivated in different ways across the age groups. Whereas emerging adults and young adults seemed to have emphasised hard work and putting effort into difficult activities, middle-aged adults seemed to have emphasised self-knowledge and setting life goals (Sotgiu et al., 2019).

These findings imply that EWB, as operationalised in the QEWB, may operate differently across age groups, which may influence the psychometric properties of the QEWB across different age groups. The findings suggest that practitioners, such as psychologists and counsellors, must consider the developmental phase of clients when considering and assessing clients’ levels of eudaimonic well-being. The QEWB shows potential for use in practice in student samples, but not the current adult sample, to measure and evaluate levels of EWB. Future research is indicated that explores EWB and the measurement thereof from a developmental perspective and more studies are needed to see if the findings replicate.

### Limitations and Recommendations

The study provide preliminary support for applying the bifactor ESEM model to QEWB data from student samples. However, the study has limitations. Firstly, the use of non-probability samples limits the generalisation of the results to other student and adult groups. Secondly, the unbiased SRMR fit statistic was calculated for the CFA and bifactor CFA models only, and not the ESEM and bifactor ESEM models since fitting these models with the lavaan package is still in its infancy. The unbiased SRMR has shown superiority to other fit indices (Ximénez et al., 2022) and future research should explore the performance of this fit statistic when ESEM and bifactor ESEM models are applied. Thirdly, the different models were tested in the same samples, and item 9 was removed from the three-factor bifactor-ESEM model for Sample 3, a result that may not hold across samples or populations as model modifications followed a data driven approach and the results may be partly or entirely influenced by idiosyncratic sample characteristics (MacCallum et al., 1992). In this sense, the current study should be conceived as a substantive illustration to explore the performance of the different analytic procedures, rather than a validation study. Future research should test the different analytical models in representative independent samples to determine its validity across samples and populations. Fourthly, while we consider the results for the adult sample to be noteworthy, especially since this study was one of very few to evaluate the performance of the QEWB among adults, the sample size was small and multicultural, and findings may not replicate in other adult samples. Future research should investigate the performance of the QEWB in other larger adult groups to not only determine to what extent the scale is usable in adult samples, but also to better understand how the underlying theoretical construct manifest and operate across age groups. Such research may be done from a developmental perspective where item functioning in various developmental phases are investigated, while cultural/contextual variables are also considered.

## Conclusion

The dimensionality of the QEWB and its underlying theoretical construct has been a contentious issue in the literature. The current study supports previous findings that EWB is multidimensional, but at the same time represents an overarching higher order construct, and suggests that analytic models that allow for the articulation of this structure are preferred when modelling the QEWB. The study further found support for configural invariance of the scale across three language versions of the scale completed by university students, with the samples representing more African and more Western cultural groups. However, metric and scalar invariance were not achieved. Although factor variances, covariances, and mean scores cannot be compared, the findings imply that there are similarities in how EWB manifests and is expressed across cultural groups. For the adult sample, use of the QEWB in the current sample is not recommended. The QEWB seems to show differential psychometric properties for different developmental phases which points towards the need to validate, and establish the equivalence of, the QEWB in age groups other than emerging adults. This also suggests the broader need for investigation of the manifestation of EWB across different age groups, and suggest that practitioners should take cognisance of possible varying manifestations of EWB in different developmental phases.

## Data Availability Statement

The data analysed in this study is subject to the following licenses/restrictions: The datasets generated during and/or analysed during the current study are available from the author MW on reasonable request, subject to ethics approval. Requests to access these datasets should be directed to MW, Marie.Wissing@nwu.ac.za.

## Ethics Statement

The studies involving human participants were reviewed and approved by the Health Research Ethics Committee of the North-West University, South Africa, ethics approval number: NWU 00002-07-A2. The patients/participants provided their written informed consent to participate in this study.

## Author Contributions

AC, LS, and MW contributed to the design and planning of the study. LS and MW were responsible for the gathering and capturing of the data. AC, LS, and WS attended to the statistical analyses and the interpretation of the results. AC drafted the manuscript, incorporated the suggestions from the co-authors, and prepared the final manuscript for submission. WS drafted selected parts of the Data Analysis and Results sections. LS and MW provided continuous and critical feedback regarding the intellectual content of the document. The final manuscript was read and approved by all authors.

## Conflict of Interest

The authors declare that the research was conducted in the absence of any commercial or financial relationships that could be construed as a potential conflict of interest.

## Publisher’s Note

All claims expressed in this article are solely those of the authors and do not necessarily represent those of their affiliated organizations, or those of the publisher, the editors and the reviewers. Any product that may be evaluated in this article, or claim that may be made by its manufacturer, is not guaranteed or endorsed by the publisher.

## Footnotes

1 Before removal of item 9 from Models 4a and 5a, the residual variances were specified to be larger than zero but this resulted in a first-order derivative matrix that was not positive-definite.
