# Supplementary material for: Further Investigation of the Dimensionality of the Questionnaire for Eudaimonic Well-Being
Source: Front Psychol. 2022 May 6;13:795770. doi: 10.3389/fpsyg.2022.795770 (PMC9121013; doi:10.3389/fpsyg.2022.795770)
Supplement: Supplementary file 1 [file Table_1.docx]

Table S1

*Descriptive Statistics of the Individual Items of the QEWB for All Samples*

|  | Sample 1: QEWB-English (Students) | | | |  | Sample 2: QEWB-Afrikaans (Students) | | | |  | Sample 3: QEWB-Setswana (Students) | | | |  | Sample 4: QEWB-English (Adults) | | | |
| --- | --- | --- | --- | --- | --- | --- | --- | --- | --- | --- | --- | --- | --- | --- | --- | --- | --- | --- | --- |
| Item | *M* | *SD* | Skew | Kurt |  | *M* | *SD* | Skew | Kurt |  | *M* | *SD* | Skew | Kurt |  | *M* | *SD* | Skew | Kurt |
| 1 | 4.80 | 1.63 | -0.54 | -0.23 |  | 4.84 | 1.33 | -0.31 | -0.32 |  | 4.50 | 1.55 | -0.23 | -0.36 |  | 5.14 | 1.48 | -0.71 | 0.18 |
| 2 | 5.02 | 1.75 | -0.79 | -0.13 |  | 4.80 | 1.53 | -0.58 | -0.24 |  | 5.16 | 1.63 | -0.75 | -0.16 |  | 5.36 | 1.38 | -0.88 | 0.66 |
| 3 | 3.99 | 2.03 | 0.04 | -1.17 |  | 4.64 | 1.83 | -0.32 | -0.98 |  | 3.25 | 2.05 | 0.47 | -1.08 |  | 3.83 | 1.98 | 0.20 | -1.14 |
| 4 | 5.72 | 1.37 | -1.09 | 0.77 |  | 5.60 | 1.22 | -0.84 | 0.43 |  | 5.07 | 1.80 | -0.67 | -0.64 |  | 5.46 | 1.36 | -1.06 | 1.09 |
| 5 | 6.15 | 1.29 | -1.85 | 3.36 |  | 5.85 | 1.41 | -1.31 | 1.12 |  | 5.72 | 1.58 | -1.31 | 1.21 |  | 5.86 | 1.22 | -1.13 | 1.17 |
| 6 | 5.60 | 1.53 | -1.39 | 1.64 |  | 5.48 | 1.24 | -0.79 | 0.56 |  | 5.78 | 1.35 | -1.19 | 1.29 |  | 5.57 | 1.24 | -0.77 | 0.20 |
| 7 | 5.53 | 1.80 | -1.13 | 0.16 |  | 5.21 | 1.56 | -0.62 | -0.48 |  | 4.53 | 1.95 | -0.20 | -1.15 |  | 5.22 | 1.83 | -0.74 | -0.70 |
| 8 | 6.06 | 1.19 | -1.52 | 2.48 |  | 4.75 | 1.41 | -0.39 | -0.08 |  | 5.44 | 1.55 | -0.93 | 0.25 |  | 5.67 | 1.13 | -0.86 | 0.59 |
| 9 | 4.85 | 1.81 | -0.68 | -0.45 |  | 4.07 | 1.70 | -0.19 | -0.86 |  | 4.71 | 1.67 | -0.58 | -0.24 |  | 5.12 | 1.61 | -0.93 | 0.31 |
| 10 | 4.64 | 2.10 | -0.47 | -1.11 |  | 4.75 | 1.66 | -0.41 | -0.69 |  | 4.90 | 1.96 | -0.59 | -0.86 |  | 4.86 | 1.80 | -0.68 | -0.48 |
| 11 | 5.24 | 1.93 | -0.84 | -0.55 |  | 4.84 | 1.75 | -0.52 | -0.69 |  | 5.44 | 1.94 | -0.88 | -0.66 |  | 5.07 | 1.99 | -0.58 | -1.10 |
| 12 | 5.82 | 1.71 | -1.53 | 1.34 |  | 5.70 | 1.51 | -1.22 | 0.86 |  | 5.08 | 1.99 | -0.64 | -0.92 |  | 5.10 | 1.91 | -0.61 | -0.99 |
| 13 | 5.92 | 1.21 | -1.22 | 1.69 |  | 5.63 | 1.19 | -1.01 | 1.32 |  | 5.56 | 1.50 | -0.85 | -0.06 |  | 5.30 | 1.33 | -1.04 | 1.37 |
| 14 | 5.28 | 1.47 | -1.03 | 0.96 |  | 5.42 | 1.17 | -0.92 | 1.00 |  | 4.86 | 1.48 | -0.38 | -0.36 |  | 5.38 | 1.22 | -0.75 | 0.45 |
| 15 | 6.28 | 1.21 | -2.35 | 6.42 |  | 5.90 | 1.11 | -1.36 | 2.55 |  | 5.63 | 1.60 | -1.18 | 0.59 |  | 5.89 | 1.25 | -1.38 | 2.18 |
| 16 | 4.55 | 2.19 | -0.33 | -1.37 |  | 4.37 | 1.95 | -0.19 | -1.26 |  | 4.40 | 2.08 | -0.20 | -1.23 |  | 5.04 | 1.97 | -0.62 | -0.93 |
| 17 | 4.95 | 1.65 | -0.76 | 0.06 |  | 5.63 | 1.18 | -0.87 | 0.44 |  | 5.19 | 1.62 | -0.84 | 0.12 |  | 5.09 | 1.42 | -0.68 | 0.21 |
| 18 | 6.01 | 1.24 | -1.85 | 4.06 |  | 5.72 | 1.20 | -1.27 | 2.33 |  | 5.78 | 1.39 | -1.11 | 0.53 |  | 5.67 | 1.21 | -0.71 | -0.19 |
| 19 | 5.72 | 1.80 | -1.46 | 1.13 |  | 6.01 | 1.30 | -1.73 | 2.97 |  | 5.18 | 1.95 | -0.62 | -0.96 |  | 5.32 | 1.84 | -0.77 | -0.71 |
| 20 | 4.79 | 1.86 | -0.48 | -0.87 |  | 4.86 | 1.68 | -0.55 | -0.61 |  | 3.89 | 2.10 | 0.15 | -1.36 |  | 4.86 | 1.90 | -0.47 | -1.15 |
| 21 | 4.91 | 1.85 | -0.62 | -0.66 |  | 4.49 | 1.65 | -0.38 | -0.57 |  | 5.72 | 1.60 | -1.26 | 0.73 |  | 5.30 | 1.57 | -0.99 | 0.49 |

*Note*. QEWB = Questionnaire for Eudaimonic Well-being; *M* = mean; *SD* = standard deviation; Skew = skewness; Kurt = kurtosis.
