# Supplementary material for: Further Investigation of the Dimensionality of the Questionnaire for Eudaimonic Well-Being
Source: Front Psychol. 2022 May 6;13:795770. doi: 10.3389/fpsyg.2022.795770 (PMC9121013; doi:10.3389/fpsyg.2022.795770)
Supplement: Supplementary file 2 [file Table_2.DOCX]

**Table S2**

*Multivariate Skewness and Kurtosis*

| Sample | Skewness | | |  | Kurtosis | | |
| --- | --- | --- | --- | --- | --- | --- | --- |
|  | *b*_1,_ *_p_* | *z*_1,_ *_p_* | *p*_skew_ |  | *b*_2,_ *_p_* | *z*_2,_ *_p_* | *p*_kurt_ |
| 1 | 118.2 | 5890.09 | <.001 |  | 672.6 | 52.74 | <.001 |
| 2 | 61.61 | 4774.91 | <.001 |  | 585.58 | 35.59 | <.001 |
| 3 | 85.93 | 2964.53 | <.001 |  | 540.36 | 13.28 | <.001 |
| 4 | 167.28 | 2481.28 | <.001 |  | 514.79 | 4.83 | <.001 |

*Note. b*_1,_ *_p_ =* Mardia’s skewness; *z*_1,_ *_p_* = test statistic of Mardia’s skewness; *p*_skew_ = *p*-value of the test statistic of Mardia’s skewness; *b*_2,_ *_p_ =* Mardia’s kurtosis; *z*_2,_ *_p_* = test statistic of Mardia’s kurtosis; *p*_kurt_ = *p*-value of the test statistic of Mardia’s kurtosis.
