# Supplementary material for: Further Investigation of the Dimensionality of the Questionnaire for Eudaimonic Well-Being
Source: Front Psychol. 2022 May 6;13:795770. doi: 10.3389/fpsyg.2022.795770 (PMC9121013; doi:10.3389/fpsyg.2022.795770)
Supplement: Supplementary file 3 [file Table_3.DOCX]

**Table S3**

*Inter-item Correlations of the QEWB-English for Student Sample 1*

| Item 1 | | 2 | 3 | | 4 | 5 | | | 6 | 7 | | 8 | 9 | | 10 | | | 11 | | 12 | 13 | | 14 | 15 | | 16 | | 17 | | 18 | | 19 | | | 20 | 21 | |  |
| --- | --- | --- | --- | --- | --- | --- | --- | --- | --- | --- | --- | --- | --- | --- | --- | --- | --- | --- | --- | --- | --- | --- | --- | --- | --- | --- | --- | --- | --- | --- | --- | --- | --- | --- | --- | --- | --- | --- |
| 1 | 1 |  |  |  | | |  |  | | |  |  | |  | |  |  | |  | | |  |  | |  | |  | |  | |  | |  |  | | |  | |
| 2 | .307 | 1 |  |  | | |  |  | | |  |  | |  | |  |  | |  | | |  |  | |  | |  | |  | |  | |  |  | | |  | |
| 3 | .035 | -.010 | 1 |  | | |  |  | | |  |  | |  | |  |  | |  | | |  |  | |  | |  | |  | |  | |  |  | | |  | |
| 4 | .304 | .412 | .110 | 1 | | |  |  | | |  |  | |  | |  |  | |  | | |  |  | |  | |  | |  | |  | |  |  | | |  | |
| 5 | .170 | .226 | .113 | .420 | | | 1 |  | | |  |  | |  | |  |  | |  | | |  |  | |  | |  | |  | |  | |  |  | | |  | |
| 6 | .342 | .417 | .056 | .307 | | | .376 | 1 | | |  |  | |  | |  |  | |  | | |  |  | |  | |  | |  | |  | |  |  | | |  | |
| 7 | .072 | .119 | .181 | .089 | | | .245 | .205 | | | 1 |  | |  | |  |  | |  | | |  |  | |  | |  | |  | |  | |  |  | | |  | |
| 8 | .248 | .182 | -.024 | .417 | | | .261 | .180 | | | .056 | 1 | |  | |  |  | |  | | |  |  | |  | |  | |  | |  | |  |  | | |  | |
| 9 | .341 | .581 | .001 | .342 | | | .244 | .472 | | | .102 | .181 | | 1 | |  |  | |  | | |  |  | |  | |  | |  | |  | |  |  | | |  | |
| 10 | -.008 | -.107 | -.021 | .033 | | | .131 | .051 | | | -.035 | .114 | | .003 | | 1 |  | |  | | |  |  | |  | |  | |  | |  | |  |  | | |  | |
| 11 | .160 | .381 | .077 | .236 | | | .171 | .364 | | | .232 | .108 | | .398 | | -.107 | 1 | |  | | |  |  | |  | |  | |  | |  | |  |  | | |  | |
| 12 | .083 | .105 | .087 | .135 | | | .112 | .013 | | | .254 | .080 | | .055 | | .018 | .156 | | 1 | | |  |  | |  | |  | |  | |  | |  |  | | |  | |
| 13 | .179 | .210 | .059 | .515 | | | .321 | .258 | | | .081 | .421 | | .176 | | .106 | .113 | | .125 | | | 1 |  | |  | |  | |  | |  | |  |  | | |  | |
| 14 | .248 | .207 | -.098 | .343 | | | .298 | .272 | | | .194 | .190 | | .252 | | .049 | .169 | | .144 | | | .308 | 1 | |  | |  | |  | |  | |  |  | | |  | |
| 15 | .213 | .233 | .047 | .238 | | | .237 | .526 | | | .282 | .326 | | .327 | | .173 | .285 | | .078 | | | .339 | .328 | | 1 | |  | |  | |  | |  |  | | |  | |
| 16 | .186 | .186 | .116 | .117 | | | .068 | .273 | | | .254 | .054 | | .219 | | -.054 | .209 | | .119 | | | -.020 | .067 | | .139 | | 1 | |  | |  | |  |  | | |  | |
| 17 | .247 | .242 | .048 | .253 | | | .171 | .285 | | | .034 | .248 | | .244 | | .075 | .034 | | .089 | | | .278 | .181 | | .255 | | .129 | | 1 | |  | |  |  | | |  | |
| 18 | .148 | .111 | -.035 | .201 | | | .171 | .246 | | | .152 | .379 | | .153 | | .180 | .030 | | .137 | | | .442 | .236 | | .379 | | .025 | | .354 | | 1 | |  |  | | |  | |
| 19 | .044 | .041 | .264 | .134 | | | .090 | .132 | | | .246 | .108 | | .043 | | -.038 | .277 | | .292 | | | .112 | .081 | | .170 | | .207 | | .013 | | .136 | | 1 |  | | |  | |
| 20 | .315 | .213 | .175 | .183 | | | .188 | .275 | | | .223 | .095 | | .183 | | -.119 | .254 | | .241 | | | .088 | .163 | | .117 | | .378 | | .197 | | .086 | | .351 | 1 | | |  | |
| 21 | .218 | .507 | -.034 | .263 | | | .076 | .366 | | | .101 | .141 | | .496 | | .127 | .302 | | .075 | | | .054 | .247 | | .195 | | .284 | | .189 | | .049 | | -.081 | .172 | | | 1 | |
