# Supplementary material for: Further Investigation of the Dimensionality of the Questionnaire for Eudaimonic Well-Being
Source: Front Psychol. 2022 May 6;13:795770. doi: 10.3389/fpsyg.2022.795770 (PMC9121013; doi:10.3389/fpsyg.2022.795770)
Supplement: Supplementary file 4 [file Table_4.DOCX]

**Table S4**

*Inter-item Correlations of the QEWB-Afrikaans for Student Sample 2*

| Item 1 | | 2 | 3 | 4 | 5 | 6 | 7 | 8 | 9 | 10 | 11 | 12 | 13 | 14 | 15 | 16 | 17 | 18 | 19 | 20 | 21 |
| --- | --- | --- | --- | --- | --- | --- | --- | --- | --- | --- | --- | --- | --- | --- | --- | --- | --- | --- | --- | --- | --- |
| 1 | 1 |  |  |  |  |  |  |  |  |  |  |  |  |  |  |  |  |  |  |  |  |
| 2 | .396 | 1 |  |  |  |  |  |  |  |  |  |  |  |  |  |  |  |  |  |  |  |
| 3 | .078 | .032 | 1 |  |  |  |  |  |  |  |  |  |  |  |  |  |  |  |  |  |  |
| 4 | .284 | .230 | .159 | 1 |  |  |  |  |  |  |  |  |  |  |  |  |  |  |  |  |  |
| 5 | .185 | .185 | .079 | .152 | 1 |  |  |  |  |  |  |  |  |  |  |  |  |  |  |  |  |
| 6 | .339 | .345 | .106 | .287 | .346 | 1 |  |  |  |  |  |  |  |  |  |  |  |  |  |  |  |
| 7 | .073 | .130 | .177 | .035 | .099 | .116 | 1 |  |  |  |  |  |  |  |  |  |  |  |  |  |  |
| 8 | .237 | .198 | .123 | .097 | .103 | .237 | -.021 | 1 |  |  |  |  |  |  |  |  |  |  |  |  |  |
| 9 | .374 | .562 | .106 | .233 | .182 | .361 | .074 | .196 | 1 |  |  |  |  |  |  |  |  |  |  |  |  |
| 10 | .139 | .112 | .032 | .140 | .123 | .117 | .047 | .079 | .110 | 1 |  |  |  |  |  |  |  |  |  |  |  |
| 11 | .175 | .323 | .167 | .167 | .142 | .168 | .192 | .055 | .480 | .051 | 1 |  |  |  |  |  |  |  |  |  |  |
| 12 | .177 | .190 | .218 | .213 | .125 | .211 | .127 | .148 | .250 | .063 | .365 | 1 |  |  |  |  |  |  |  |  |  |
| 13 | .195 | .197 | .099 | .248 | .161 | .290 | .087 | .138 | .232 | .180 | .113 | .126 | 1 |  |  |  |  |  |  |  |  |
| 14 | .269 | .315 | .017 | .243 | .171 | .299 | .097 | .172 | .287 | .133 | .135 | .178 | .284 | 1 |  |  |  |  |  |  |  |
| 15 | .175 | .130 | .056 | .160 | .194 | .340 | .061 | .153 | .204 | .165 | .058 | .197 | .298 | .373 | 1 |  |  |  |  |  |  |
| 16 | .173 | .303 | .130 | .143 | .064 | .225 | .149 | .180 | .324 | .050 | .318 | .160 | .073 | .115 | .032 | 1 |  |  |  |  |  |
| 17 | .320 | .220 | .132 | .291 | .235 | .329 | .081 | .230 | .252 | .081 | .211 | .249 | .308 | .349 | .301 | .114 | 1 |  |  |  |  |
| 18 | .278 | .136 | .024 | .172 | .218 | .200 | .032 | .139 | .163 | .230 | .095 | .150 | .340 | .284 | .327 | -.003 | .405 | 1 |  |  |  |
| 19 | .082 | .116 | .214 | .094 | .113 | .203 | .219 | .130 | .153 | -.066 | .178 | .221 | .024 | .044 | .054 | .199 | .191 | .025 | 1 |  |  |
| 20 | .187 | .240 | .202 | .192 | .145 | .239 | .232 | .131 | .229 | -.022 | .282 | .227 | .134 | .105 | .160 | .227 | .099 | .003 | .226 | 1 |  |
| 21 | .375 | .517 | .069 | .260 | .183 | .343 | .065 | .185 | .641 | .150 | .470 | .239 | .175 | .264 | .212 | .407 | .320 | .128 | .170 | .220 | 1 |
