# Supplementary material for: Further Investigation of the Dimensionality of the Questionnaire for Eudaimonic Well-Being
Source: Front Psychol. 2022 May 6;13:795770. doi: 10.3389/fpsyg.2022.795770 (PMC9121013; doi:10.3389/fpsyg.2022.795770)
Supplement: Supplementary file 5 [file Table_5.DOCX]

**Table S5**

*Inter-item Correlations of the QEWB-Setswana for Student Sample 3*

| Item 1 | | 2 | 3 | 4 | 5 | 6 | 7 | 8 | 9 | 10 | 11 | 12 | 13 | 14 | 15 | 16 | 17 | 18 | 19 | 20 | 21 |
| --- | --- | --- | --- | --- | --- | --- | --- | --- | --- | --- | --- | --- | --- | --- | --- | --- | --- | --- | --- | --- | --- |
| 1 | 1 |  |  |  |  |  |  |  |  |  |  |  |  |  |  |  |  |  |  |  |  |
| 2 | .334 | 1 |  |  |  |  |  |  |  |  |  |  |  |  |  |  |  |  |  |  |  |
| 3 | -.087 | -.073 | 1 |  |  |  |  |  |  |  |  |  |  |  |  |  |  |  |  |  |  |
| 4 | .323 | .377 | -.158 | 1 |  |  |  |  |  |  |  |  |  |  |  |  |  |  |  |  |  |
| 5 | .045 | .033 | -.147 | .180 | 1 |  |  |  |  |  |  |  |  |  |  |  |  |  |  |  |  |
| 6 | .214 | .352 | -.093 | .217 | .224 | 1 |  |  |  |  |  |  |  |  |  |  |  |  |  |  |  |
| 7 | -.128 | .105 | .009 | .100 | .150 | .060 | 1 |  |  |  |  |  |  |  |  |  |  |  |  |  |  |
| 8 | .119 | .116 | -.197 | .222 | .233 | .301 | .100 | 1 |  |  |  |  |  |  |  |  |  |  |  |  |  |
| 9 | .102 | .502 | -.019 | .274 | -.084 | .191 | .079 | .256 | 1 |  |  |  |  |  |  |  |  |  |  |  |  |
| 10 | .082 | .163 | -.204 | .047 | .117 | .196 | .044 | .203 | .228 | 1 |  |  |  |  |  |  |  |  |  |  |  |
| 11 | .112 | .139 | .047 | .056 | .132 | .184 | .250 | .227 | -.005 | .158 | 1 |  |  |  |  |  |  |  |  |  |  |
| 12 | .029 | -.001 | -.019 | .118 | .199 | .056 | .204 | .184 | -.082 | .079 | .449 | 1 |  |  |  |  |  |  |  |  |  |
| 13 | .237 | .323 | -.160 | .316 | .143 | .353 | .206 | .384 | .168 | .292 | .202 | .200 | 1 |  |  |  |  |  |  |  |  |
| 14 | .411 | .295 | -.216 | .306 | .192 | .294 | .005 | .246 | .125 | .166 | .062 | -.018 | .334 | 1 |  |  |  |  |  |  |  |
| 15 | .119 | .264 | -.075 | .209 | .253 | .357 | .157 | .232 | .173 | .163 | .203 | .101 | .342 | .259 | 1 |  |  |  |  |  |  |
| 16 | .118 | .035 | .131 | -.036 | .018 | .017 | .112 | .074 | -.019 | .018 | .315 | .165 | .006 | -.004 | -.010 | 1 |  |  |  |  |  |
| 17 | .299 | .341 | -.165 | .231 | .077 | .274 | .154 | .264 | .212 | .174 | .175 | .068 | .243 | .210 | .223 | .059 | 1 |  |  |  |  |
| 18 | .201 | .279 | -.059 | .356 | .174 | .427 | .221 | .340 | .159 | .147 | .175 | .153 | .429 | .339 | .478 | .009 | .385 | 1 |  |  |  |
| 19 | .034 | .146 | .069 | .052 | .164 | .057 | .289 | .175 | .079 | .113 | .502 | .475 | .192 | .030 | .169 | .258 | .203 | .184 | 1 |  |  |
| 20 | -.096 | .002 | .039 | .012 | .152 | .025 | .214 | .150 | -.006 | .086 | .247 | .318 | .064 | -.195 | .105 | .210 | .041 | .042 | .286 | 1 |  |
| 21 | .214 | .360 | -.121 | .319 | .284 | .418 | .093 | .317 | .302 | .225 | .216 | .161 | .394 | .319 | .347 | .042 | .238 | .327 | .219 | .022 | 1 |
