# Supplementary material for: Further Investigation of the Dimensionality of the Questionnaire for Eudaimonic Well-Being
Source: Front Psychol. 2022 May 6;13:795770. doi: 10.3389/fpsyg.2022.795770 (PMC9121013; doi:10.3389/fpsyg.2022.795770)
Supplement: Supplementary file 6 [file Table_6.DOCX]

**Table S6**

*Inter-item Correlations of the QEWB-English for Adult Sample 4*

| Item 1 | | 2 | 3 | 4 | 5 | 6 | 7 | 8 | 9 | 10 | 11 | 12 | 13 | 14 | 15 | 16 | 17 | 18 | 19 | 20 | 21 |
| --- | --- | --- | --- | --- | --- | --- | --- | --- | --- | --- | --- | --- | --- | --- | --- | --- | --- | --- | --- | --- | --- |
| 1 | 1 |  |  |  |  |  |  |  |  |  |  |  |  |  |  |  |  |  |  |  |  |
| 2 | .244 | 1 |  |  |  |  |  |  |  |  |  |  |  |  |  |  |  |  |  |  |  |
| 3 | .020 | .050 | 1 |  |  |  |  |  |  |  |  |  |  |  |  |  |  |  |  |  |  |
| 4 | .245 | .492 | .076 | 1 |  |  |  |  |  |  |  |  |  |  |  |  |  |  |  |  |  |
| 5 | .124 | .273 | .176 | .225 | 1 |  |  |  |  |  |  |  |  |  |  |  |  |  |  |  |  |
| 6 | .214 | .447 | .039 | .421 | .304 | 1 |  |  |  |  |  |  |  |  |  |  |  |  |  |  |  |
| 7 | .004 | .125 | .342 | .159 | .289 | .266 | 1 |  |  |  |  |  |  |  |  |  |  |  |  |  |  |
| 8 | .265 | -.032 | -.088 | .067 | .241 | .106 | .046 | 1 |  |  |  |  |  |  |  |  |  |  |  |  |  |
| 9 | .098 | .503 | .106 | .403 | .076 | .307 | .036 | -.044 | 1 |  |  |  |  |  |  |  |  |  |  |  |  |
| 10 | -.019 | -.051 | .069 | .030 | .022 | .006 | -.019 | .046 | -.034 | 1 |  |  |  |  |  |  |  |  |  |  |  |
| 11 | -.018 | .406 | .382 | .285 | .230 | .304 | .439 | -.046 | .380 | -.078 | 1 |  |  |  |  |  |  |  |  |  |  |
| 12 | .025 | -.060 | .235 | .007 | .163 | .041 | .318 | -.010 | -.097 | .047 | .367 | 1 |  |  |  |  |  |  |  |  |  |
| 13 | .291 | .077 | .055 | .184 | .220 | .183 | .053 | .307 | .123 | .070 | .098 | .158 | 1 |  |  |  |  |  |  |  |  |
| 14 | .198 | .355 | -.084 | .180 | .278 | .208 | .001 | .019 | .141 | -.107 | .054 | .142 | .172 | 1 |  |  |  |  |  |  |  |
| 15 | .120 | .092 | .111 | .148 | .268 | .256 | .381 | .372 | .023 | .093 | .232 | .222 | .292 | .140 | 1 |  |  |  |  |  |  |
| 16 | .091 | .273 | .271 | .173 | .072 | .335 | .445 | -.046 | .282 | -.074 | .631 | .337 | .082 | .012 | .260 | 1 |  |  |  |  |  |
| 17 | .242 | .146 | .005 | .240 | .174 | .387 | .104 | .092 | .240 | .138 | .091 | .005 | .229 | .244 | .197 | .097 | 1 |  |  |  |  |
| 18 | .190 | .157 | -.045 | .190 | .235 | .320 | .234 | .282 | .025 | .208 | .154 | .282 | .273 | .214 | .447 | .121 | .416 | 1 |  |  |  |
| 19 | .157 | .138 | .228 | .102 | .324 | .165 | .389 | .070 | -.099 | .054 | .318 | .415 | .069 | .124 | .383 | .409 | .039 | .194 | 1 |  |  |
| 20 | .147 | .124 | .144 | .252 | .129 | .240 | .327 | .053 | .039 | .114 | .357 | .275 | .054 | .096 | .358 | .349 | .123 | .359 | .561 | 1 |  |
| 21 | .214 | .542 | -.032 | .530 | .042 | .418 | .106 | -.078 | .563 | -.078 | .311 | -.011 | .064 | .264 | -.005 | .220 | .289 | .095 | .021 | .210 | 1 |
